# Use of Valacyclovir for the treatment of cytomegalovirus antigenemia after hematopoietic stem cell transplantation

**DOI:** 10.1186/s12878-015-0028-2

**Published:** 2015-06-19

**Authors:** Shin-Yeu Ong, Ha-Thi-Thu Truong, Colin Phipps Diong, Yeh-Ching Linn, Aloysius Yew-Leng Ho, Yeow-Tee Goh, William Ying-Khee Hwang

**Affiliations:** Department of Hematology, Singapore General Hospital, Singapore, Singapore

## Abstract

**Background:**

Valacyclovir has been used for prophylaxis against cytomegalovirus (CMV) infection after hematopoietic stem cell transplantation (HSCT). We investigated the efficacy and safety of high-dose Valacyclovir as pre-emptive therapy in patients with CMV antigenemia after HSCT.

**Methods:**

In a retrospective single center study of 61 patients, we compared the rates of viral clearance, recurrent antigenemia and adverse events in patients with pp65 CMV antigenemia who received high dose Valacyclovir (n = 15), Valganciclovir (n = 16), and Foscarnet (n = 30).

**Results:**

Overall, 60/61 (98 %) of cases achieved CMV antigenemia clearance by day 28, and no patient developed CMV disease. After adjusting for age, sex, diagnosis, CMV serological status, donor type, CMV antigen level, graft-versus-host disease (GVHD) therapy, and conditioning regimen, there were no significant differences in the rates of viral clearance at day 14 in patients who received Valganciclovir (0.18, 95 % confidence interval (CI) 0.01 to 2.15, p = 0.17) and Foscarnet (OR 0.22, 95 % CI 0.03 to 2.40, p = 0.22), compared with Valacyclovir (assigned OR = 1.00). Recurrent antigenemia by day 180 after clearance of the initial CMV episode occurred in 34/61 (56 %) of patients. Using the multivariate model adjusting for the same covariates, there were also no significant differences in secondary episodes of CMV between treatment groups. With regards to adverse effect monitoring, Foscarnet led to significantly increased creatinine levels (*P* = 0.009), while Valganciclovir led to significant decrease in neutrophil counts (*P* = 0.012).

**Conclusion:**

High dose Valacyclovir is a potential alternative to Valganciclovir and Foscarnet in the stable post-HSCT patient who has cytopenia and is not keen for inpatient treatment of CMV antigenemia.

## Background

Cytomegalovirus (CMV) infection poses a serious clinical challenge to hematopoietic stem cell transplant (HSCT) patients as it can result in numerous complications associated with significant morbidity and mortality. These include retinitis, encephalitis, pneumonia, hepatitis and gastrointestinal ulceration. Two approaches for the prevention of CMV infection are currently practiced. The first is universal prophylaxis with routine administration of an antiviral agent to all patients after transplant. Benefits of prophylaxis is that monitoring may not be required if an effective antiviral is used, but some patients are exposed to drug-related toxicities unnecessarily. The second approach is pre-emptive therapy, that is initiated when CMV infection is detected, but before the development of CMV-associated symptoms. Pre-emptive therapy depends on early detection of CMV in blood, which is aided by the ready availability of pp65 antigenemia and DNA PCR-based assays. Both approaches are equally effective in preventing CMV disease.

Ganciclovir, and its modified oral formulation, Valganciclovir (GCV), are first line agents for pre-emptive therapy against CMV [1–3]. When cytopenias are present, Foscarnet is used as an alternative. Although as effective as ganciclovir, Foscarnet is associated with renal toxicity and requires inpatient therapy, hence it is reserved as second line therapy [4, 5]. Valacyclovir satisfies several criteria for an ideal pre-emptive therapeutic agent due to its low toxicity profile, and excellent bioavailability after oral administration [6]. High dose Valacyclovir has already been shown to be safe and effective in CMV prophylaxis after solid organ and stem cell transplantation [7–13], but has not been adequately studied as an antiviral for pre-emptive therapy against CMV antigenemia. Valacyclovir is potentially an important alternative agent in patients with cytopenia who are not eligible for Ganciclovir, and who are unwilling to be hospitalized for intravenous Foscarnet.

We hypothesized that Valacyclovir could be useful as a single agent against CMV antigenemia after HSCT, without significant hematologic or renal toxicity. To evaluate this treatment approach, a retrospective cohort study comparing the use of Valacyclovir, Valganciclovir, and Foscarnet was performed in our institution. The primary outcome was viremia clearance. Secondary outcomes included recurrent antigenemia and adverse events.

## Methods

### Patients

All consecutive adult patients who underwent allogeneic bone marrow, peripheral blood stem cell, or cord blood transplantation at Singapore General Hospital between January 2008 and September 2011 were included if they had an initial episode of CMV antigenemia before a single antiviral (Valacyclovir, Valganciclovir, or Foscarnet) was started for pre-emptive therapy. Departmental practice guidelines, integrated with patients’ preferences, determined the choice of antiviral regimen. Ganciclovir is the first line pre-emptive therapy for CMV in our inpatients, while Foscarnet is used in patients with neutropenia or previous ganciclovir treatment failure. Outpatients with normal gastrointestinal absorption received Valganciclovir, while patients with neutropenia received Valacyclovir. Patients who developed CMV disease before or at the time of the initial detection of CMV antigenemia were excluded. All patients also received acyclovir for prophylaxis against varicella zoster virus and trimethoprim-sulfamethoxazole for *Pneumocystis jirovecii* prophylaxis until immunosuppression was discontinued. Prophylaxis for fungal infections was either posaconazole or itraconazole. Calcineurin-inhibitor-based therapies were the most commonly used GVHD prophylaxis regimens, with the inclusion of anti-thymocyte globulins (ATG) in unrelated donor transplants. All participants gave their informed consent for participation in the research database and the database collection was approved by the institutional review board of the Singapore General Hospital.

### Detection of CMV reactivation

All patients were screened for CMV infection using a CMV pp65 antigenemia assay at least twice in the first week after transplant, and at least once a week subsequently. The CMV antigenemia assay was performed as described previously [14], and ≥ 1 CMV antigen positive cell per million leukocytes was used as the threshold for pre-emptive therapy.

### Pre-emptive therapy

Patients were treated with either Foscarnet at 90 mg/kg twice daily (BID), or 45 mg/kg BID if creatinine clearance is less than 60 ml/min; Valganciclovir a 900 mg BID or 450 mg BID if creatinine clearance is less than 60 ml/min, or Valacyclovir 2 g four times daily (QID) or 1 g QID in the presence of renal impairment. The median duration of treatment was 14 days. Clearance of CMV antigenemia was defined as 0 positive cells per million leukocytes via the CMV pp65 antigenemia assay. The incidence of recurrent CMV antigenemia after treatment with each agent was recorded for 180 days after the clearance of an initial episode of CMV antigenemia. Patients who relapsed after successful clearance of CMV antigenemia were treated at the discretion of the physician.

### Monitoring of adverse events

Patients were monitored for the development of CMV disease as defined previously [15], as well as significant side effects of Valacyclovir, Valganciclovir, or Foscarnet. Haemograms and biochemical panels were performed at least once a week to look for neutropenia, thrombocytopenia and renal impairment. Mortality rates and causes of mortality for up to 6 months post-transplant were recorded.

### Statistical analysis

Values are expressed as median (range), and the significance of differences was determined using the chi-square test or analysis of variance, as appropriate. Some analyses compared changes in pre- and post-treatment cell counts and serum creatinine between two groups; these were analyzed using the paired t-test. Multivariable logistic regression models were used to determine the odds of viral clearance at day 14, and of recurrent antigenemia in patients treated with Valganciclovir and Foscarnet, compared with Valacyclovir. Potential confounders considered include age, gender, CMV serological status, donor type, CMV antigen level at diagnosis, conditioning regimen, and graft-versus-host disease therapy.

## Results

### Patient characteristics

The demographic characteristics of the three groups of patients are shown in Table 1. Comparisons of the three groups for parameters that could influence CMV reactivation showed significant difference with respect to age, but not CMV serological status, sex, donor type, indication for transplant and conditioning regimen. Patients requiring systemic corticosteroids or other agents (e.g. ertanercept, mycophenolate mofetil, tacrolimus) for treatment of GVHD were statistically similar between groups.

**Table 1:** Patient characteristics

		All (n = 61)	Valacyclovir (n = 15)	Valganciclovir (n = 16)	Foscarnet (n = 30)	*P* ^a^
Median age, years (range)		41 (16–66)	47 (18–61)	50 (30–57)	37 (16–66)	0.017
Male, N (%)		31 (50.8)	9 (60.0)	5 (31.3)	27 (56.7)	0.186
Diagnosis, N (%)						0.509
	Acute myeloid leukemia	30 (49.2)	7 (46.7)	9 (56.3)	14 (46.7)	
	Acute lymphoid leukemia	13 (21.3)	3 (20.0)	1 (6.3)	9 (30.0)	
	Non-Hodgkin’s lymphoma	2 (3.3)	1 (6.7)	1 (6.3)	0 (0.0)	
	Myelodysplastic syndrome	6 (9.8)	1 (6.7)	1 (6.3)	4 (13.3)	
	Others	10 (16.4)	3 (20.0)	4 (25.0)	3 (10.0)	
Conditioning regimen, N (%)						0.374
	Myeloabalative	32 (53.3)	9 (60.0)	11 (68.8)	12 (41.4)	
	Non-Myeloabalative	14 (23.3)	2 (13.3)	3 (18.8)	9 (31.0)	
	Reduced Intensity	14 (23.3)	4 (26.7)	2 (12.5)	8 (27.6)	
Donor type, N (%)						0.078
	Related	31 (50.8)	11 (73.3)	10 (62.5)	10 (33.3)	
	Unrelated	21 (34.4)	2 (13.3)	5 (31.3)	14 (46.7)	
	Cord Blood	9 (14.8)	2 (13.3)	1 (6.25)	6 (20.0)	
CMV serologic status, N (%)						0.586
	Donor-/recipient+	9 (14.8)	1 (6.7)	3 (18.8)	5 (16.7)	
	Donor+/recipient+	52 (85.3)	14 (93.3)	13 (81.3)	25 (83.3)	
GVHD (during study)						0.155
	None	27 (44.3)	5 (33.3)	4 (25.0)	18 (60.0)	
	Grade I-II	30 (49.2)	9 (60.0)	10 (62.5)	11 (36.7)	
	Grade II-IV	4 (6.6)	1 (6.7)	2 (12.5)	1 (33.3)	
GVHD treatment						0.119
	None	25 (41.0)	5 (33.3)	3 (18.8)	17 (56.7)	
	Steroids	30 (49.1)	9 (60.0)	11 (68.9)	10 (33.3)	
	Others	6 (9.8)	1 (6.7)	2 (12.5)	3 (10.0)	
Pre-treatment laboratory results, median (range)						
	Creatinine (μM)	78 (40–240)	86 (46–182)	72 (44–240)	78 (40–205)	0.601
	ANC (x10^3^/mm^3^)	2.6 (0.2-22.8)	2.4 (0.2-6.6)	3.6 (1.1-15.6)	1.7 (0.8-22.8)	0.093
	Platelet (x10^3^/mm^3^)	64 (7–260)	98 (15–197)	68 (7–244)	32.5 (8–260)	0.053

*CMV*, cytomegalovirus; *GVHD*, graft versus host disease; *ANC*, absolute neutrophil count

^a^P value for difference by treatment group, based on chi-square test or analysis of variance

**Table 2:** Response to pre-emptive CMV therapy

	All (n = 61)	Valacyclovir (n = 15)	Valganciclovir (n = 16)	Foscarnet (n = 30)	*P*
Median time to antigenemia (days from transplant, range)	27 (12–387)	39 (16–387)	31.5 (15–119)	24.5 (12–104)	0.084
Median viral load (No. of CMV positive cells per million leukocytes, range)	3 (1–750)	3 (1–40)	3 (2–181)	3.5 (1–750)	0.772
Clearance, N (%)	60 (98.1)	15 (100)	16 (100)	29 (96.7)	0.591
Recurrent antigenemia, N (%)	34 (55.8)	7 (46.7)	11 (68.8)	16 (53.3)	0.434
Median days to recurrence	43.5 (11–173)	59 (27–173)	42 (14–94)	38 (11–163)	0.081

*CMV*, cytomegalovirus

### CMV antigenemia and pre-emptive treatment

The median number of CMV antigen-positive cells at the initiation of pre-emptive therapy did not differ between groups (*P* = 0.77), and the median viral load for all included patients was 3 (range 1 to 750). Overall, 60/61 (98 %) of cases achieved CMV antigenemia clearance by day 28, with no significant differences between treatment groups (p = 0.591). By day 14, clearance rates among groups who received Valacyclovir, Valganciclovir, and Foscarnet were 14/15 (93 %), 13/16 (81 %), and 22/30 (73 %) respectively. After adjusting for age, sex, diagnosis, CMV serological status, donor type, CMV antigen level, GVHD therapy, and conditioning regimen, there were no significant differences in the rates of viral clearance at day 14 in patients who received Valganciclovir (odds ratio (OR) 0.18, 95 % confidence interval (CI) 0.01 to 2.15, p = 0.17) and Foscarnet (OR 0.22, 95 % CI 0.03 to 2.40, p = 0.22), compared with Valacyclovir (assigned OR = 1.00).

Although high rates of CMV clearance were achieved, recurrent antigenemia by day 180 after clearance of the initial CMV episode occurred in 55.8 % of patients. After adjusting for the same covariates, there were no significant differences in secondary episodes of CMV among patients who received Valganciclovir (OR 3.36, 95 % CI 0.62 to 18.3, p = 0.17) and Foscarnet (OR 1.02, 95 % CI 0.20 to 5.25, p = 0.98, compared with Valacyclovir (OR = 1.00). No patients developed CMV disease during the course of the study. Response to pre-emptive CMV treatment using the three different anti-virals are detailed in Table 2.

### Adverse events and survival

The elevation in serum creatinine levels was significantly higher after treatment with Foscarnet, compared to Valacyclovir or Valganciclovir (*P* = 0.009). Treatment with Valganciclovir led to a significant decrease in neutrophil counts, compared to Foscarnet or Valacyclovir (*P* = 0.012). Changes in pre- and post-treatment platelet levels did not differ significantly between groups (Table 3). One patient died of neutropenic enterocolitis on post-transplant day 102 in the Valacyclovir group, one patient died of disease progression on post-transplant day 138 in the Valganciclovir group, and three patients died of disease progression and sepsis on post-transplant days 81, 129, and 131 in the Foscarnet group.

**Table 3:** Hematological and renal toxicity of treatment

		All (n = 61)	Valacyclovir (n = 15)	Valganciclovir (n = 16)	Foscarnet (n = 30)	*P*
Change in parameter (post-treatment – pre-treatment), median (range)						
	Creatinine (μM)	14 (−31 to 126)	2 (−31 to 57)	−2.5 (−70 to 73)	31.6 (−65 to 126)	0.009
	ANC (x10^3^/mm^3^)	−0.05 (−20.6 to 5.0)	0.19 (−4.2 to 3)	−1.5 (−7.5 to −1.1)	1.1 (−20.6 to 5.0)	0.012
	Platelet (x10^3^/mm^3^)	6 (−123 to 196)	6 (−72 to 82)	−7.5 (−123 to 196)	13 (−92 to 150)	0.335

*ANC*, Absolute neutrophil count

## Discussion

In this retrospective study, pre-emptive therapy with Valacyclovir, Valganciclovir and Foscarnet achieved high viral clearance rates in post-HSCT patients with CMV antigenemia (98 %). After adjusting for potential confounders including age, sex, CMV serotype, CMV antigen level at diagnosis, donor type, conditioning regimen and GVHD treatment, the rates of viral clearance and recurrent antigenemia were not significantly different in patients receiving high dose Valacyclovir, compared with Ganciclovir or Foscarnet. Viral clearance with Valacyclovir was achieved with significantly less reduction in neutrophil count or rise in creatinine levels. However, it is important to bear in mind that patients who received Valacyclovir in our study were discharged outpatients who were at least one month post-transplant, had no other active infection, and were not debilitated. Thus our findings may only extend to the stable post-HSCT patient.

Another limitation of our study is the use of a low threshold value of 1 CMV-positive cell per million leukocytes to start pre-emptive therapy. It has been suggested that low positive results may represent transient reactivation [16], or even a rare false positive result [17], hence clearance may in part be spontaneous. However, Boeckh *et al*. showed that the discontinuation of gancyclovir below the threshold of 3 positive cells per 50,000 leukocytes led to a risk of CMV disease [18]. When a single positive cell is used as trigger, the rate of CMV disease was reduced [19]. Other investigators have also used a single positive cell as trigger, recognizing that even low viral loads might be significant in the HSCT patient [16, 20, 21]. Given the rapid doubling time of CMV in immunosuppressed paients [22], withholding anti-CMV therapy may pose significant risks. Future studies applying a higher threshold to begin pre-emptive therapyin a larger group of patients are needed to confirm the therapeutic effect of Valacyclovir in high-level CMV antigenemia.

To our knowledge, no previous report has investigated the use of Valacyclovir for pre-emptive therapy of CMV antigenemia, although several randomized and retrospective studies have demonstrated the efficacy of Valacyclovir as prophylactic therapy. For example, results from large randomized multicenter studies have shown that Valacyclovir is more effective in preventing CMV antigenemia than oral acyclovir [11], and has similar efficacy as Ganciclovir in preventing CMV infection and disease [12]. Similarly, retrospective reports have established the potential benefit of Valacyclovir as a prophylactic agent against CMV reactivation, compared with no or other forms of CMV prophylaxis, with significantly reduced rates and delay of CMV reactivation [10, 13]. The small sample size in our study precludes definitive conclusions about the efficacy of Valacyclovir as pre-emptive therapy in HSCT patients, but results are encouraging. Importantly, Valacyclovir represents a cost effective alternative to Valganciclovir [23].

Limitations of this study include its retrospective study design, non-randomized treatment allocation, and small sample size. In recent years, more institutions have switched from pp65 antigenemia assays to quantitative PCR methods to guide pre-emptive therapy. PCR based methods are rapid, more sensitive, provide more precise quantitation of CMV, and can be used in patients with severe neutropenia. Its disadvantage includes inter-assay and inter-laboratory variability in viral load reporting, which has complicated attempts to standardize thresholds for initiating and stopping pre-emtive therapy. Recently, the WHO international reference standard was developed, which enables uniform viral load reporting and interpretation. It remains to be studied if the viral load threshold for preemptive therapy using Valacyclovir is comparable to that using Valganciclovir or Foscarnet.

## Conclusions

In conclusion, pre-emptive Valacyclovir, Foscarnet and Valacyclovir led to similar clearance of CMV antigenemia and rates of recurrence. High dose Valacyclovir is potentially a safe and cost-effective option for pre-emptive treatment of CMV antigenemia in the stable post-HSCT patient who has cytopenia or prefers outpatient treatment. These findings must be interpreted in light of limitations inherent to retrospective observational studies. Further prospective randomized studies are needed to validate the efficacy suggested by the results of this retrospective study.
